# A public mid‐density genotyping platform for cultivated cranberry (*Vaccinium macrocarpon* Aiton)

**DOI:** 10.1002/tpg2.70118

**Published:** 2025-10-08

**Authors:** Shufen Chen, Meng Lin, Cristiane H. Taniguti, Xuemei Tang, Fernando de la Torre, Gina M. Sideli, Massimo Iorizzo, Patrick P. Edger, Jeffrey Neyhart, Juan Zalapa, Nahla Bassil, Kasia Heller‐Uszynska, Dongyan Zhao, Craig T. Beil, Moira J. Sheehan

**Affiliations:** ^1^ Cornell Institute of Biotechnology Breeding Insight Cornell University Ithaca New York USA; ^2^ USDA‐ARS, Cranberry Genetics and Genomics Laboratory, Vegetable Crops Research Unit, Department of Plant and Agroecosystem Sciences Madison Wisconsin USA; ^3^ USDA‐ARS Chatsworth New Jersey USA; ^4^ Department of Horticultural Science North Carolina State University Kannapolis North Carolina USA; ^5^ Department of Horticulture Michigan State University East Lansing Michigan USA; ^6^ Genetic Improvement for Fruits & Vegetables Laboratory USDA‐ARS Chatsworth New Jersey USA; ^7^ USDA‐ARS National Clonal Germplasm Repository Corvallis Oregon USA; ^8^ Diversity Arrays Technology University of Canberra Bruce Australian Capital Territory Australia

## Abstract

Cranberry (*Vaccinium macrocarpon* Aiton), a native North American fruit crop, has gained global popularity due to its unique flavor and health benefits. As the market expands for new cranberry products, the requirements to produce varieties that meet new standards have increased. DNA‐assisted breeding in cranberry has been limited due to the lack of cost‐effective genotyping tools. To address this gap, Breeding Insight developed and validated a 3K DArTag panel. Target loci were strategically selected from 507K single‐nucleotide polymorphisms (SNPs), generated from resequencing 53 diverse cultivated cranberry accessions. Selection criteria prioritized even genomic distribution, genic regions, maximum genetic diversity among North American breeding germplasm, and markers associated with known quantitative trait loci. The cranberry 3K DArTag panel was validated using a diverse collection of cranberry accessions, interspecific hybrids, and two F_1_ populations. The panel, optimized for cultivated *V*. *macrocarpon*, demonstrated a high average amplification rate (83.4%) and robust performance in its close relatives, *Vaccinium microcarpum* and *Vaccinium oxycoccos*, as well as somewhat lower but acceptable performance in interspecific hybrids. However, transferability to the more distant blueberry was limited. The panel successfully revealed expected ploidy levels and population structure among the tested materials. Two individual linkage maps and one consensus map were constructed for the mapping populations, with an average marker density of 0.68 markers per centimorgan. This cost‐effective (∼$15/sample), rapid genotyping platform offers valuable capabilities for public and private breeding programs. Its open‐access nature enables genetic datasets generated from the marker panel to be compared and integrated across projects and geographical boundaries.

## Core Ideas

- Cranberry is an important crop for human health and nutrition.
- Genomics‐assisted methods have been underutilized in cranberry breeding.
- Creation of a cranberry 3K DArTag panel provides affordable access to breeders.
- Genomic data and Qploidy can assess genome ploidy in mixed ploidy populations.
- The mid‐density panel hastens data‐driven selections in cranberry crop breeding.

## Plain Language Summary

Cranberries are a vital crop for human health and nutrition, yet few genomic tools exist to enhance breeding and selection for improved cranberry varieties. Here, we describe the creation of one such genomic tool: a DArTag genotyping marker panel that targets variants at 3000 sites across the cranberry genome. We demonstrate that data from this marker panel can elucidate genomic relationships, including distinguishing samples with various genome‐duplication levels (ploidies), identifying mislabeled accessions, and creating linkage maps from biparental populations. The DArTag panel offers several advantages important to cranberry breeders, including fast and affordable genotyping, SNP and microhaplotypes data outputs, and a more flexible platform for adding and removing markers as needed. The public release of this genomic tool lowers the barrier to entry for breeders in obtaining and using genomic data in cranberry breeding and crop improvement efforts.

**Abbreviations**
Alt: alternative allele
BLASTn: basic local alignment search tool (nucleotide to nucleotide)
FASTQ: FAST‐All with quality score
GBS: genotyping‐by‐sequencing
GWAS: genome‐wide association study
HWE: Hardy–Weinberg equilibrium
IBS: identity by state
LG (or lg): linkage group
MADC: missing allele discovery counts
MAF: minor allele frequency
MAS: marker‐assisted selection
NCBI: National Center for Biotechnology Information
PC: principal component
PCA: principal component analysis
PIC: polymorphism information content
QTL: quantitative trait locus or loci
Ref: reference allele
RF: recombination fraction
SNP: single‐nucleotide polymorphism
SSR: simple sequence repeat

## 1 INTRODUCTION

The American cranberry (*Vaccinium macrocarpon* Aiton) is a native fruit crop of North America, which was foraged and utilized by the Wompanoag people ∼8000 years before the first European settlers rediscovered cranberries (called “crane berry” for its flower morphology) in the 1600s (Massachusetts Cranberry, https://www.cranberries.org/history). The first report of cranberry cultivation dates to 1816 in Massachusetts (Eck, 1990). Cranberry is a relatively recent crop, and many cultivars still in production today are “wild selections” (Vorsa & Zalapa, 2019). Cranberries grow naturally in sandy, nutrient‐poor, and acidic soils with high water tables and northern temperate climates as long trailing vines close to the ground (Kloet, 1988). Commercial production mimics the natural environment by cultivating large areas with walled berms (marshes) that allow for controlled flooding during the harvest, where berries are floated and harvested for processing in autumn (Eck, 1990; Vorsa & Zalapa, 2019).

Cranberry is experiencing an increase in global popularity in both production and consumption, attributed to its distinct flavor profile and potential health benefits (Gallardo et al., 2018; Nemzer et al., 2022; Vorsa & Zalapa, 2019). The United States, followed by Canada, is the largest cranberry‐producing country worldwide. In 2024, cranberry production in the United States reached approximately 8.9 million barrels (~447,300 tons), marking a 9% increase from the approximately 8.1 million barrels (~405,500 tons produced in 2023 (Cranberry Total Production, USDA NASS 2025; https://downloads.usda.library.cornell.edu/usda‐esmis/files/zs25x846c/mc87rn20c/w37656321/ncit0525.pdf). The value of cranberry production reached around 311 million US dollars in 2024, experiencing a surge of more than 9% between 2022 and 2024 (Cranberry Value of Production, USDA NASS, 2025; https://downloads.usda.library.cornell.edu/usda‐esmis/files/zs25x846c/mc87rn20c/w37656321/ncit0525.pdf). Americans consume an estimated 400 million pounds of cranberries annually, with 20% of that consumption occurring during November and December. Additionally, the per capita consumption of cranberries in the United States is 2.3 pounds, mainly in the form of juice or juice blends, with minor consumption as fresh or dried (AgMRC, 2024; https://www.agmrc.org/commodities‐products/fruits/cranberries).

The increasing consumption of cranberry products and those with aded cranberry has spurred a demand for new cultivars that align with the needs of cranberry growers, processors, and consumers (Gallardo et al., 2018; Vorsa & Zalapa, 2019). Traditionally, cranberry varieties have been developed primarily through phenotypic evaluation, focusing on fruit characteristics (yield, color intensity, berry size, whitish waxy coating, ripening season, seed number, coloring in storage, and shelf‐life quality) and vegetative traits (upright length, leaf shape, and leaf size) (Gallardo et al., 2018; Vorsa & Johnson‐Cicalese, 2012; Vorsa & Zalapa, 2019). Phenotypic evaluation and selection for cranberry cultivar breeding is a lengthy process, requiring 3 years from pollination crosses to juvenile flowering, and an additional 6–8 years after field planting for comprehensive phenotypic assessment (Vorsa & Zalapa, 2019). The North American cranberry industry faces multiple challenges, including increasing disease and insect pressures, which are likely exacerbated by climate change, coupled with new restrictions on the use of insecticides and fungicides (Johnson‐Cicalese et al., 2015). To address these challenges, cranberry breeding programs that aim to develop disease‐ and pest‐resistant varieties with a range of berry quality phenotypes have become increasingly important (Gallardo et al., 2018; Vorsa & Johnson‐Cicalese, 2012; Vorsa & Zalapa, 2019).

Molecular techniques have been extensively employed for nearly four decades to enhance the efficiency and precision of breeding programs in major staple food crops like tomato, maize, and barley (Feuerstein et al., 1990; Hasan et al., 2021; Helentjaris et al., 1985; Tanksley, 1983). The development and implementation of molecular techniques in cranberry have been slow, despite the integration of genomic‐based techniques being shown to significantly expedite the breeding process, improve resistance traits, and better meet the industry's needs (Vorsa & Zalapa, 2019). Cranberry's diploid nature, modest genome size (∼492 Mbp), and the development of new genomic tools facilitated the development of marker‐based breeding strategies in cranberry (Diaz‐Garcia et al., 2021). These assets include genome assemblies (Diaz‐Garcia et al., 2021; Kawash et al., 2022; Polashock et al., 2014), linkage maps based on single sequence repeat (SSR) and single‐nucleotide polymorphism (SNP) markers (Covarrubias‐Pazaran et al., 2016, Georgi et al., 2013; Schlautman et al., 2017, 2015), identification of quantitative trait loci (QTL) associated with agronomic traits (Daverdin et al., 2017; Diaz‐Garcia, Covarrubias‐Pazaran, et al., 2018; Diaz‐Garcia, Schlautman, et al., 2018; Fong et al., 2020; Georgi et al., 2013; Maule et al., 2024), and genomic selection studies (Covarrubias‐Pazaran et al., 2018). Most of these prior SNP datasets were generated using the genotyping‐by‐sequencing (GBS) approach, which requires minimal prior knowledge of polymorphism rates (Elshire et al., 2011). However, GBS, as a routine genotyping platform, presents several issues for breeders, including high missing data rates, amplification from random sites, the requirement for high‐quality DNA, the high cost of producing datasets, the need for specialized bioinformatics skills in data processing, and the challenges associated with combining datasets.

**Core Ideas**

- Cranberry is an important crop for human health and nutrition.
- Genomics‐assisted methods have been underutilized in cranberry breeding.
- Creation of a cranberry 3K DArTag panel provides affordable access to breeders.
- Genomic data and Qploidy can assess genome ploidy in mixed ploidy populations.
- The mid‐density panel hastens data‐driven selections in cranberry crop breeding.

Modern breeding programs have largely moved away from GBS as a routine genotyping platform for many of the reasons stated above. SNP arrays and chips, as well as kompetative allele‐specific PCR (KASP) assays, offer a faster and more cost‐effective method for generating genotypic data at the same loci in all samples, facilitating easier data processing for breeders. Similar to SNP arrays, newer targeted‐amplicon‐based genotyping technologies such as DArTag (Diversity Array Technology—DArT), Flex‐Seq (RAPiD Genomics), and Capture‐Seq (LGC Genomics) have low missing data rates and query the same exact loci in all samples across genotyping projects, allowing for new data to be easily appended to existing data (Darrier et al., 2019; Telfer et al., 2019; Wang et al., 2020). The amount of data returned is in the tens of thousands, rather than the millions of reads from GBS, which simplifies downstream bioinformatics processing (Darrier et al., 2019; Milner et al., 2019; Telfer et al., 2019; Wang et al., 2020). This, in turn, accelerates the analysis for marker‐assisted selection (MAS), introgression tracking, linkage mapping, genome‐wide association studies (GWAS), and genomic prediction (Darrier et al., 2019). Despite these opportunities, a mid‐density platform that is cost‐effective and that can be used as a routine tool in cranberry is still not available.

To fill this gap, we developed and validated a novel 3K DArTag marker panel comprising 3059 genome‐wide loci. We thoroughly tested the panel's efficacy across diverse materials varying in ploidy and origin, including interspecific hybrid progeny between cranberry and blueberry, to confirm the panel's applicability beyond cultivated cranberry. Furthermore, we validated the panel's utility for linkage mapping and QTL studies using two bi‐parental F_1_ populations. This work delivers an affordable and robust genetic resource to the cranberry breeding community, facilitating molecular breeding and genomic prediction applications.

## 2 MATERIALS AND METHODS

### 2.1 Performance of cranberry samples on a blueberry 3K DArTag panel

Given the genetic similarity between blueberry and cranberry, a set of 343 cranberry lines (Table 1; Table S1) was genotyped using the 3K blueberry DArTag panel (BberryDArTagBICU; Zhao et al., 2024) to evaluate cross‐species marker transferability. We evaluated the commercially cultivated species, American cranberry (*V. macrocarpon*) and its closest wild relative (*Vaccinium microcarpum*), which are both naturally diploid (2*n* = 2*x* = 24) along with a wild tetraploid cranberry species *Vaccinium oxycoccos* (Diaz‐Garcia et al., 2021). Therefore, the 343 tested cranberry accessions included 183 *V. macrocarpon* (2*x*) diverse cranberries, 11 *V. microcarpum* (2*x*) selfed samples, 10 *V. oxycoccos* (4*x*) samples, two colchicine‐generated *V. macrocarpon* tetraploid samples, and one *V. macrocarpon* sample derived from a cross between other colchicine‐generated tetraploid parents, and two diploid F_1_ populations (*V. macrocarpon*)—CNJ16‐41 (reciprocal crosses of NJS98‐18 and CNJ99‐9‐96, *n* = 64) and CNJ16‐45 (reciprocal crosses of NJS98‐18 and CNJ97‐105‐4, *n* = 69), along with their three parents (NJS98‐18, CNJ99‐9‐96, and CNJ97‐105‐4) (Table 1). DArT provided the read count report for the 3000 target loci. To evaluate the detection rate of the marker panel, marker loci with <10 reads across the 343 lines were considered failures of amplification. Sample‐level missing rates were evaluated across the entire population and within each of the three groups. Minor allele frequency (MAF) (Table S2) was calculated for each of the 3000 markers using the ratio of minor allele read counts to total read counts. A locus was considered polymorphic if its minor allele read count ratio was greater than or equal to the frequency of a singleton (1/number of gametes), corresponding to 0.14% across all 343 accessions.

**TABLE 1:** Population composition of the testing materials for the 3K blueberry and cranberry DArTag panels.

Testing material	Samples tested on 3K blueberry DArTag panel	Samples tested on 3K cranberry DArTag panel
F_1_ ^a^	136	136
*V. macrocarpon* (diverse; 2*x*)	183	183
*V. macrocarpon* (colchicine‐generated; 4*x*)^b^	3	4
*V. microcarpum* (selfed; 2*x*)	11	11
*V. oxycoccos* (4*x*)^b^	10	18
Interspecific hybrids (4*x*)^c^	–	18
Blueberry (4*x*)	–	2
Total	343	372

^a^ F_1_ population includes CNJ16‐41 (reciprocal crosses of NJS98‐18 and CNJ99‐9‐96), CNJ16‐45 (reciprocal crosses of NJS98‐18 and CNJ97‐105‐4), and their parents (NJS98‐18, CNJ99‐9‐96, and CNJ97‐105‐4).

^b^ Nine additional accessions were added to the 13 autotetraploid accessions (*V. oxycoccos* and colchicine‐generated *V. macrocarpon*) tested in the blueberry DArTag panel, including one colchicine‐generated accession (father of one of the blueberry × cranberry crosses) and eight autotetraploid accessions, of which four were putative mothers of another cranberry × blueberry cross.

^c^ Interspecific hybrids consisted of 18 blueberry × cranberry and cranberry × blueberry hybrid progenies from two interspecific crosses.

### 2.2 SNP discovery and selection of 3K loci for a cranberry DArTag genotyping panel

A cranberry 3K DArTag panel was developed from a diverse panel of 53 cranberry accessions (Table S3). These 53 accessions were selected from a panel of 192 diverse lines used for resequencing in the Vaccinium Coordinated Agricultural Project (Yocca et al., 2023). The selection included 24 frequently used cranberry breeding cultivars and 29 additional lines chosen to maximize genetic diversity using the CDmean method (Rincent et al., 2012). We aligned the 507,124 raw SNP markers discovered from the cranberry resequencing data (Clare et al., 2025) to the *V. macrocarpon* cv. Stevens v1.0 genome (Diaz‐Garcia et al., 2021) to obtain a high‐confidence set of 10,861 SNPs (Figure S1, marker selection pipelines). Retention in this set required SNPs to have (1) a quality score >20; (2) a minor allele frequency greater than 5%; (3) a missing rate <75%; (4) a nonsignificant Hardy–Weinberg equilibrium (HWE) test value at *p*‐value of 0.01; and (5) even genomic distribution.

DArTag is a hybridization/amplicon‐based targeted genotyping platform developed by DArT (Blyton et al., 2023; https://www.diversityarrays.com/services/targeted‐genotying/). Oligos were custom‐designed to target known genetic variants (SNPs and insertions/deletions of less than 20 bp) and their flanking genomic regions, resulting in sequencing products of 81 bp in length. The 10,861 SNPs mentioned above were submitted to DArT for quality control, from which 2937 SNPs were selected by targeting even genomic distribution, prioritizing gene coding and QTL regions, and minimizing gaps across the genome. Additionally, 122 markers potentially associated with agronomic traits were also included in the panel. Oligos for a total of 3059 loci were designed at DArT and synthesized by Integrated DNA Technologies (Coralville, IA, USA).

### 2.3 Genotyping results of DArTag validation samples

The cranberry 3K DArTag panel (CranDArTagBICUv1) was evaluated using the original 343 accessions used to test the blueberry 3K DArTag panel (BberryDArTagBICU; Zhao et al., 2024), along with nine additional autotetraploid accessions including eight *V. oxycoccos* samples and one (*V. macrocarpon*) sample derived from a cross between other colchicine‐generated tetraploid parents, two blueberry accessions, and 18 interspecific hybrid accessions derived from crosses between five autotetraploid cranberry accessions and two blueberry accessions (Table 1; Table S1). The interspecific hybrids were included to test the panel's cross‐species amplification capability. Thus, a total of 372 samples were genotyped and had data returned in the “missing allele discovery count” (MADC) file, which encompassed all microhaplotypes (81 bp short sequences) discovered for the 3K marker loci. These microhaplotypes contain target SNPs (used in assay design) in addition to off‐target SNPs within the amplicon. In this study, only target SNP sites were analyzed using the Ref (complete reference match) and Alt (reference match with only one variant at the target SNP site) microhaplotypes. To simplify variant calling from partially overlapping microhaplotypes, only one target SNP was retained within each 15 bp window, resulting in the removal of 353 marker loci (Table S4) from the raw MADC file. The remaining 2706 marker loci in the MADC file (Data S1) were used for downstream analysis.

### 2.4 Cranberry 3K DArTag marker detection rate

To evaluate the SNP detection rate of the 3K DArTag loci, genotype data points were set to missing when there were <10 reads (Ref + Alt) per marker per individual. The threshold of ≥10 reads was determined according to the general sequencing depth (>30) of the DArTag platform and to guarantee confident genotype calling for the downstream analysis. The sample‐level missing rate was calculated as the ratio of missing loci to total tested loci (*n* = 2706). Two F_1_ samples were removed due to their high missing rate (≥95%) (Table S5). A total of 163 SNPs were removed due to their high missing rates (≥95%) (the ratio of missing data to the remaining 370 samples), resulting in 2543 SNPs (Table S6).

### 2.5 Evaluation of sample ploidy levels

Ploidy levels of 370 validation samples were evaluated using read depth information from 2543 markers using the R package Qploidy v0.0.1(Taniguti et al., 2025). Qploidy employs a standardization method to allele count ratios ([alternative read depth]/[total read depth]) to facilitate the comparison of copy numbers across a sample's genome based on the number of heterozygous classes. This process relies on a set of reference samples with known ploidy levels for accurate standardization and ploidy estimation. Here, all samples (*n* = 370) and their tentative genotype dosages were used as references for the initial Qploidy analysis. Tentative genotype dosages were estimated using the “norm” model in the R package updog v2.1.5 (Gerard et al., 2018) with ploidy level set to 2 based on the predominantly diploid population (89%). Following initial ploidy estimation, the second Qploidy analysis utilized 135 confirmed diploid samples as references for the standardization process. Most of the test materials were estimated to have the same ploidy levels as denoted by the breeders, with the exception of two samples (CNJ16‐52‐21 and CNJ16‐50‐63) from the F_1_ populations and two colchicine‐generated accessions (Macro4x_01 and Macro4x_04). These two F_1_ accessions were expected to be diploids given their diploid parents but were found to be tetraploids, and thus were removed from the downstream analysis (Table S5).

### 2.6 Principal component analysis

A principal component analysis (PCA) was performed using read count data from 2543 markers for the 368 tested samples with QPloidy‐validated ploidy levels. The ratio of the Ref read depth to the Total (Ref + Alt) read depth was used to approximate allele frequency, and PCA was performed using the estimated allele frequency matrix using the AddPCA function in polyRAD v2.0.0 (Clark et al., 2019) and visualized using the R package ggplot2 v3.5.1 (Wickham, 2016).

### 2.7 Genotype dosage calling

Genotype dosage calling was performed in each of the five sub‐groups (F_1_ progeny from CNJ16‐45, F_1_ progeny from CNJ16‐41, diploid accessions [*V. macrocarpon* and *V. microcarpum*], autotetraploid accessions [*V. oxycoccos* and colchicine‐generated *V. macrocarpon*], and interspecific hybrids). To ensure each marker contains enough information for dosage calling within a tested panel, markers (n_CNJ16‐45 =_ 95, n_CNJ16‐41 =_ 115, n_2x =_ 3, n_4x =_ 33, n_hybrid =_ 109) with high missing rates (≥95%) were removed (Table S6). Genotype dosage calling was performed for target SNP markers using the “multidog” function in the R package updog v2.1.5 (Gerard et al., 2018). The “norm” model was implemented with ploidy levels set to 2 and 4 for the diploid and autotetraploid accessions, respectively. For two F_1_ populations, the “f1” model was applied with specified parentage samples, with the ploidy level set to 2.

Due to varying levels of sequence homology between the blueberry and cranberry genomes, DArTag markers could be amplified in either the blueberry or the cranberry sub‐genome, or in both sub‐genomes for the Blueberry × Cranberry or Cranberry × Blueberry hybrids. Amplification patterns of each 3K DArTag locus across two blueberry and five autotetraploid cranberry samples that are parents of hybrids were used to characterize them as either sub‐genome‐specific or non‐specific markers (i.e., those aligning to both sub‐genomes). If the total read counts (Ref + Alt) of a DArTag locus were ≤10 across the two blueberry samples and >10 across the five autotetraploid cranberry samples, the marker was classified as cranberry sub‐genome specific. Conversely, a marker was considered blueberry sub‐genome‐specific if it had >10 reads across the blueberry samples but ≤10 reads in the cranberry samples. A DArTag locus was considered ‘successful’ if it was amplified in both genomes with >10 reads in both cranberry and blueberry samples. A total of 2037 DArTag loci were found to have genome‐specific amplification (cranberry or blueberry), while 390 loci were amplified in both genomes. Genotype dosages of these loci were called using the “norm” model with the ploidy level set to 1 and 4, respectively, in the hybrids. Additionally, seven DArTag loci were removed because they did not have >10 reads in the cranberry and blueberry parental samples.

After dosage calling, SNP genotype dosages were filtered for their estimated allele bias (0.05 < bias  < 2), estimated proportion of individuals misclassified (prop_mis < 0.1), and the estimated overdispersion parameter (od < 0.05). A total of 2382 and 2355 SNPs were retained for CNJ16‐45 and CNJ16‐41 populations, respectively. Additionally, a total of 2412 SNPs were retained for the diploid F_1_ accessions, 2027 for autotetraploid accessions, and 2300 (1954 diploid and 346 autotetraploid SNPs) for the interspecific hybrids. Identity by state (IBS) was estimated as the proportion of shared genotype data points between a pair of samples that are important *V. macrocarpon* (2*x*) cultivars, using non‐missing SNP loci in both samples to evaluate genotype repeatability.

An SNP was considered informative if it had at least two different genotypes present among the evaluated accessions. Polymorphism information content (PIC) was calculated for all informative SNPs in the diploid diverse *V. macrocarpon* accessions, diploid *V. microcarpum* accessions, autotetraploid accessions (*V. oxycoccos*), and interspecific hybrids using the following formula described by Botstein et al. (1980):

$$\mathrm{PIC}=1-\sum_{i=1}^{n}p_{i}^{2}-\sum_{i=1}^{n-1}\sum_{j=i+1}^{n}2p_{i}^{2}p_{j}^{2},$$

where *n* is the number of alleles, $p_{i}\;$ is frequency of the *i*th allele, and $p_{j}$ is frequency of the *j*th allele.

### 2.8 Linkage map construction

Linkage maps were constructed for each of the two F_1_ populations using MAPpoly2 v2.0.0 (Mollinari & Garcia, 2019). To further clean up the data, two lines (CNJ16‐45‐26 and CNJ16‐45‐24) from the CNJ16‐45 population and three lines (CNJ16‐41‐31, CNJ16‐50‐16, and CNJ16‐50‐18) from the CNJ16‐41 population were removed, due to their relatively large genetic difference from the main clusters of their respective populations (Figure S2). Additionally, 26 overlapping markers were removed from both populations, as these markers exhibited different genotype dosages for the shared parent (NJS98‐18) in these two populations. A total of 2356 SNPs from the CNJ16‐45 population (*n* = 64) and 2329 SNPs from the CNJ16‐41 population (*n* = 60) were used for the initial analysis. We removed several marker types from subsequent analysis: non‐conforming markers (n_CNJ16‐45 =_ 1439 and n_CNJ16‐41  =_ 1489), including monomorphic markers (n_CNJ16‐45 =_ 1341 and n_CNJ16‐41 =_ 1421) and those (n_CNJ16‐45 =_ 98 and n_CNJ16‐41 =_ 68) with unexpected segregation patterns (e.g., aa × bb, bb × aa segregation), and redundant markers (n_CNJ16‐45 =_ 164 and n_CNJ16‐41 =_ 158). Additionally, markers (n_CNJ16‐45 =_ 29 and n_CNJ16‐41 =_ 24) with >10% missing rate in both populations or those deviating from the Mendelian segregation ratio (Bonferroni adjusted *p*‐value ≤ 0.05) were excluded. Pairwise recombination frequencies were computed for the remaining markers (n_CNJ16‐45 =_ 724 and n_CNJ16‐41 =_ 658) in each of the two F_1_ populations using the “*pairwise_rf*” function. Based on the recombination fraction (RF) matrices, markers (n_CNJ16‐45 =_ 32 and n_CNJ16‐41 =_ 26) that are unlikely to be linked or linked to too many regions across the genome were removed using the “*rf_filter*” function. When markers were ordered based on expected physical positions in the reference genome, some showed weak genetic linkage with the flanking markers. To address this, a marker was removed from its reference‐assigned chromosomes if it exhibited weak linkage (RF > 0.4) with at least one of its neighboring (±5) markers. If the same marker showed a strong linkage (RF < 0.1) with markers on a different chromosome, its position was manually reassigned to that chromosome. These repositioned markers do not have an exact physical location in their new chromosome because their placement was based solely on linkage information. A total of 28 (n_CNJ16‐45 =_ 20 and n_CNJ16‐41 =_ 23 with 15 overlapping markers) markers were repositioned in the two F_1_ populations. Subsequently, all remaining markers (n_CNJ16‐45 =_ 692 and n_CNJ16‐41 =_ 632) were clustered into 12 linkage groups based on the repositioned recombination fraction matrix for each of the two F_1_ populations. For each linkage group, the “*pairwise_phasing*” function was used, together with the markers’ genomic orders (physical positions), to perform phasing. A global correction error rate of 0.05 was applied to address potential genotyping errors. To increase the marker density in the genetic maps, a joint map was constructed by combining these two genetic maps based on their haplotype information from the shared parent (NJS98‐18). The joint map was created using the “*prepare_to_integrate*” and “*estimate_consensus_map*” functions within MAPpoly2.

### 2.9 Alignment of the DArTag panel against the “Ben Lear” reference genome

Several cranberry reference genomes have been published, among which the “Ben Lear” reference genome v1.0 (Kawash et al., 2022) is the most widely used after the “Stevens” genome (Diaz‐Garcia et al., 2021). To support breeders who have been working with the “Ben Lear” genome to use the DArTag panel (developed based on the “Stevens” genome) as a genomic resource, an alignment was performed to provide genomic coordinates of the 3K DArTag loci within the “Ben Lear” genome. For each of the 3K loci, alignment was performed using a 101‐bp sequence (±50 bp from the target SNP) containing the target genomic variant against the “Ben Lear” v1.0 genome using NCBI v2.16.0 BLASTn (Johnson et al., 2008). A locus was considered successfully aligned to the “Ben Lear” genome if an aligned sequence fully covered the reference sequence without insertions or deletions and had at most three nucleotide differences compared to the reference sequence.

## 3 RESULTS

### 3.1 Cross‐species performance of the blueberry DArTag panel

The performance of the 3K blueberry DArTag marker panel was evaluated on a cranberry population of 343 accessions, including diploid accessions, autotetraploid accessions, and F_1_s (from the CNJ16‐45 and the CNJ16‐41 populations). The average sample‐level missing rate was 59.7% (ranging from 53.6% to 69.8%) across all accessions. The diploid diverse *V. macrocarpon* accessions, autotetraploid *V. macrocarpon* accessions, and F_1_ populations showed similar sample‐level missing rates (59.8%, 59.0%, and 59.9%, respectively), while the diploid *V. microcarpum* and autotetraploid *V. oxycoccos* collection showed slightly lower missing rates of 57.6% and 56.7%, respectively (Table S5; Figure S3). Of the 3K blueberry markers, 1588 (53.0%) demonstrated successful amplification (>10 reads across 343 samples) in cranberry. However, only 201 of these 1588 amplified markers were polymorphic across all 343 accessions. Although the 3K blueberry DArTag marker panel achieved moderate amplification rates for cranberry accessions, it failed to adequately capture the cranberry genetic diversity. Consequently, the development of a cranberry‐specific DArTag marker panel was necessary for cranberry breeding programs.

### 3.2 Performance of the 3K cranberry DArTag marker panel

**FIGURE 1:**
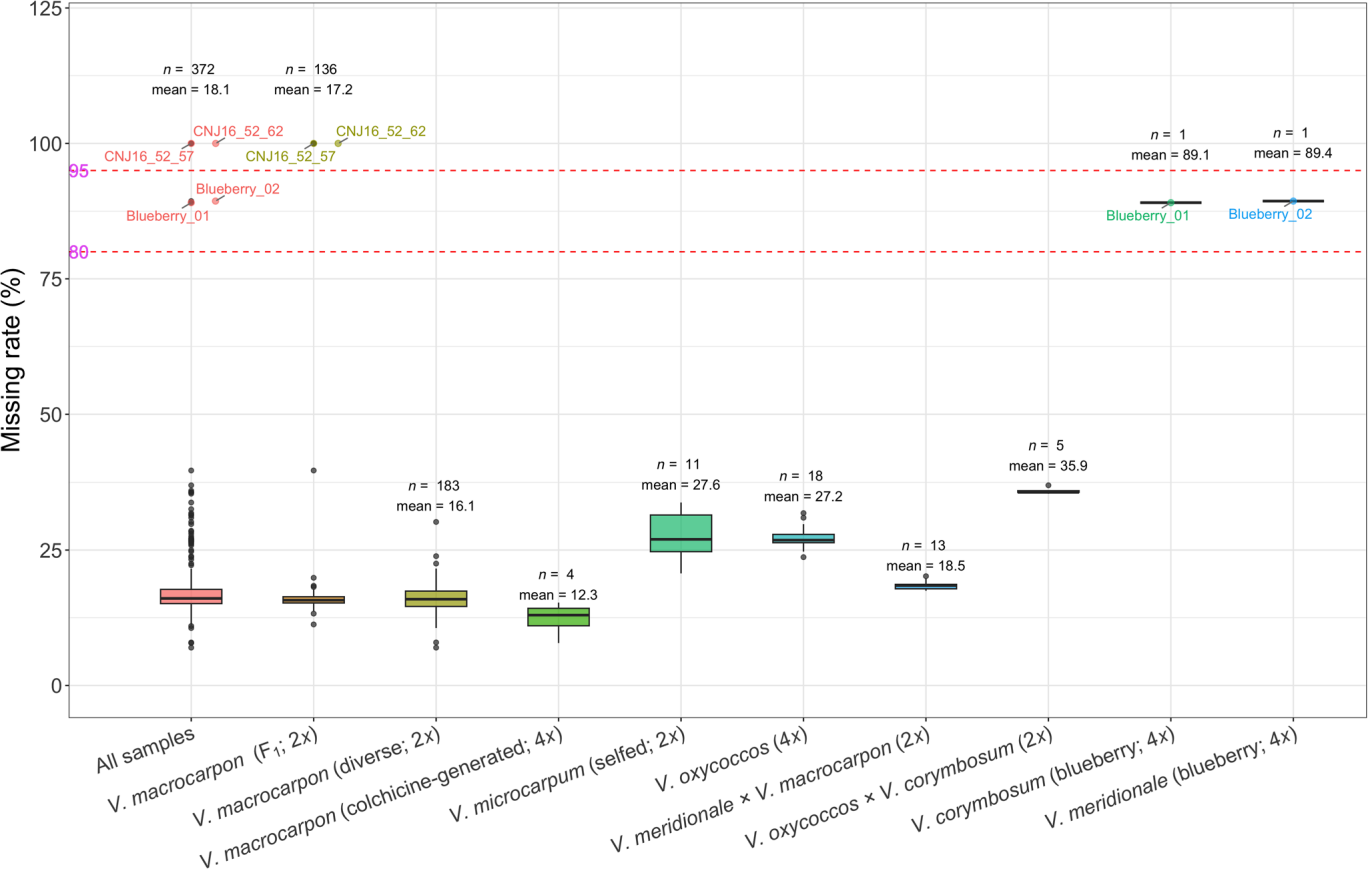
Sample‐level missing rate of tested materials for the 3K cranberry DArTag panel across all samples and in each species. For each species evaluated, “*n*” is the number of samples, and “mean” is the average missing data rate. Two dotted lines (red) indicate the 80% and 95% missing data rates, respectively. F_1_ includes CNJ16‐41 (reciprocal crosses of NJS98‐18 and CNJ99‐9‐96), CNJ16‐45 (reciprocal crosses of NJS98‐18 and CNJ97‐105‐4), and their parents (NJS98‐18, CNJ99‐9‐96, and CNJ97‐105‐4).

A total of 372 lines from different species including F_1_ progeny (*V. macrocarpon*), diploid diverse *V. macrocarpon* accessions, diploid *V. microcarpum* accessions, autotetraploid accessions (*V. oxycoccos* and colchicine‐generated *V. macrocarpon*), and artificial‐interspecific hybrids and two blueberry samples (*V. corymbosum* and *V. meridionale*) (Table S1) were used to validate the cranberry 3K DArTag panel. The average sample‐level missing rate across all samples was 18.1% (Figure 1). Each of the evaluated species showed an average sample‐level missing rate between 16.6% and 35.9%, except for two blueberry samples (89.1% and 89.4%) (Table S5). Compared to other cranberry species, the 3K cranberry panel showed consistently lower sample‐level missing rates (<18%) in the *V. macrocarpon* accessions, followed by a missing rate of 18.5% in the *V. meridionale* × *V. macrocarpon* hybrids, likely due to partial genomic compatibility inherited from *V. macrocarpon*. In contrast, the *V. oxycoccos* × *V. corymbosum* hybrids showed the highest sample‐level missing rates (35.9%), which was likely due to the greater genetic distance between *V. corymbosum*, *V. oxycoccos* (4*x*), and *V. macrocarpon* (2*x*). The diploid *V. microcarpum* and the autotetraploid *V. oxycoccos* accessions exhibited slightly elevated average missing rates of 27.6% and 27.2%, respectively, reflecting their genetic distances with *V. macrocarpon* (2*x*).

### 3.3 Genetic relationship among samples with various ploidy levels and pedigrees

**FIGURE 2:**
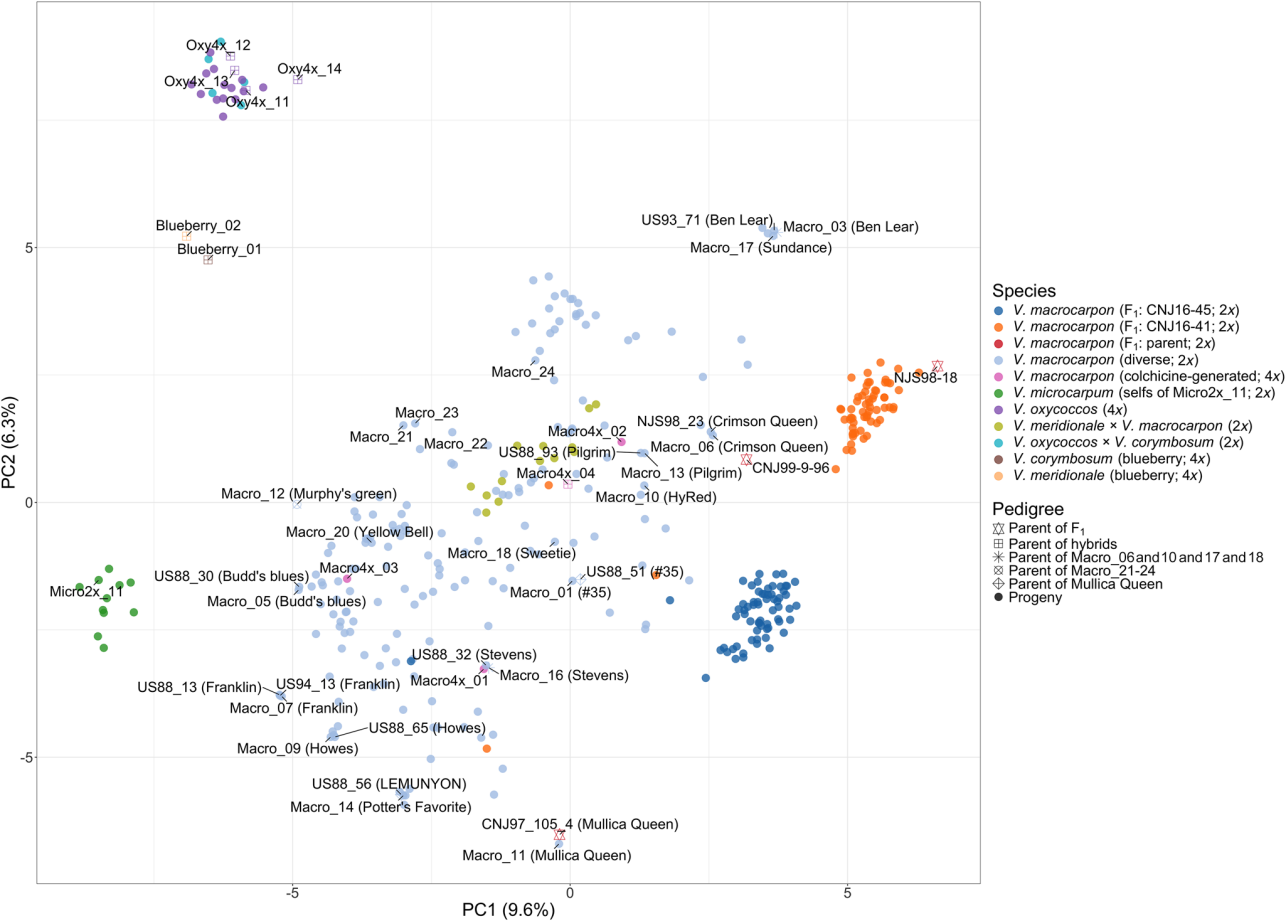
Principal component analysis (PCA) plot using read counts of 368 tested accessions. CNJ16‐45: F_1_ progeny of reciprocal crosses of NJS98‐18 and CNJ97‐105‐4; CNJ16‐41: F_1_ progeny of reciprocal crosses of NJS98‐18 and CNJ99‐9‐96.

To assess the genetic diversity and population structure in the tested materials, a PCA (Table S7) was performed using read counts. The first two principal components (PCs) explained 15.9% (PC1 9.6% and PC2 6.3%) of the total genetic variance among the tested materials (Figure 2). The two F_1_ populations (CNJ16‐45 and CNJ16‐41) were clustered between their respective parents, with only a few individuals (two in CNJ16‐45 and three in CNJ16‐41) grouping with the diploid diverse collections (Figure 2; Figure S2). These outlier F_1_ samples, possibly resulted from pollen contamination, were excluded from the linkage map construction. The 18 naturally occurring *V. oxycoccos* (4*x*) accessions and 11 *V. microcarpum* (2*x*) accessions formed distinct clusters, both of which were clearly separated from the *V. macrocarpon* (2*x*) accessions which exhibited much more diversity. A total of 14 important *V. macrocarpon* (2x) cultivars were highlighted in the PCA plot. Among these cultivars, nine of them had duplicated samples, with each pair clustering tightly in the PCA plot. Duplicated samples were used to evaluate genotype repeatability via IBS rate between each pair. The duplicated samples showed high IBS rates, ranging from 0.9888 to 1 (Table 2), indicating high genotyping consistency and supporting the reliability of this marker panel.

**TABLE 2:** Identity‐by‐state (IBS) rates for duplicated samples of important *V. macrocarpon* cultivars to assess genotyping repeatability.

Accession ID	Accession ID	IBS rate	Alias (cultivar)
Macro_01	US88_51	0.9888	#35
Macro_03	US93_71	0.9954	Ben Lear
Macro_05	US88_30	0.9959	Budd's Blues
Macro_06	NJS98_23	0.9967	Crimson Queen
Macro_07	US88_13	0.9963	Franklin
Macro_07	US94_13	0.9971	Franklin
US88‐13	US94_13	0.9967	Franklin
Macro_09	US88_65	0.9967	Howes
Macro_11	CNJ97‐105‐4	1	Mullica Queen
Macro_13	US88_93	0.9983	Pilgrim
Macro_16	US88_32	0.9996	Stevens

For non‐naturally occurred samples, their clustering patterns were largely dependent on pedigree. Five of the interspecific hybrids were clustered with their putative *V. oxycoccos* cranberry parents (Oxy4x_11, Oxy4x_12, Oxy4x_13, and Oxy4x_14), while the other 13 hybrids were clustered with their colchicine‐generated tetraploid *V. macrocarpon* parent (Macro4x_04). Similarly, four colchicine‐generated tetraploid samples (two first generation: Macro4x_01 and Macro4x_04; two second generation: Macro4x_02 and Macro4x_03) derived from *V. macrocarpon* originated parents were clustered with the rest of *V. macrocarpon* accessions. Such clustering patterns suggest that these colchicine‐generated tetraploids exhibited similar genome‐wide allele frequencies as the *V. macrocarpon* accessions, which reflects their allele and haplotype originations from diploid diverse *V. macrocarpon*.

While PCA elucidated expected population structure of the tested materials, Qploidy could estimate and validate variable sample ploidy levels using reduced prior knowledge of reference population ploidy. Highly consistent ploidy levels were observed for all diploid samples and most of the autotetraploid samples between Qploidy output and ploidy information from breeders. For colchicine‐generated tetraploid samples, the first‐generation samples (Macro4x_01 and Macro4x_04) were successfully distinguished from the second‐generation samples (Macro4x_02 and Macro4x_03 were) using Qploidy based on their unique allelic segregation patterns across the genome (Figure S4). However, neither PCA nor Qploidy was able to correctly determine the ploidy level of the first generation of colchicine‐generated samples (Macro4x_01, Macro4x_04). This limitation stems from the unique nature of the first‐generation colchicine‐induced tetraploids. While natural tetraploids display five possible allele dosages (0–4) and diploids show three (0, 1, and 2), first‐generation colchicine‐induced tetraploids contain an auto‐duplicated genome that maintains diploid‐like dosage patterns (0, 1, and 2). As a result, these samples can be mistakenly classified as diploids despite having tetraploid genome content. Overall, the clustering pattern from PCA aligns with the expected population structure and supports the effectiveness of the marker panel in detecting and representing genetic relationships in the tested samples.

### 3.4 Panel effectiveness in extant accessions

**FIGURE 3:**
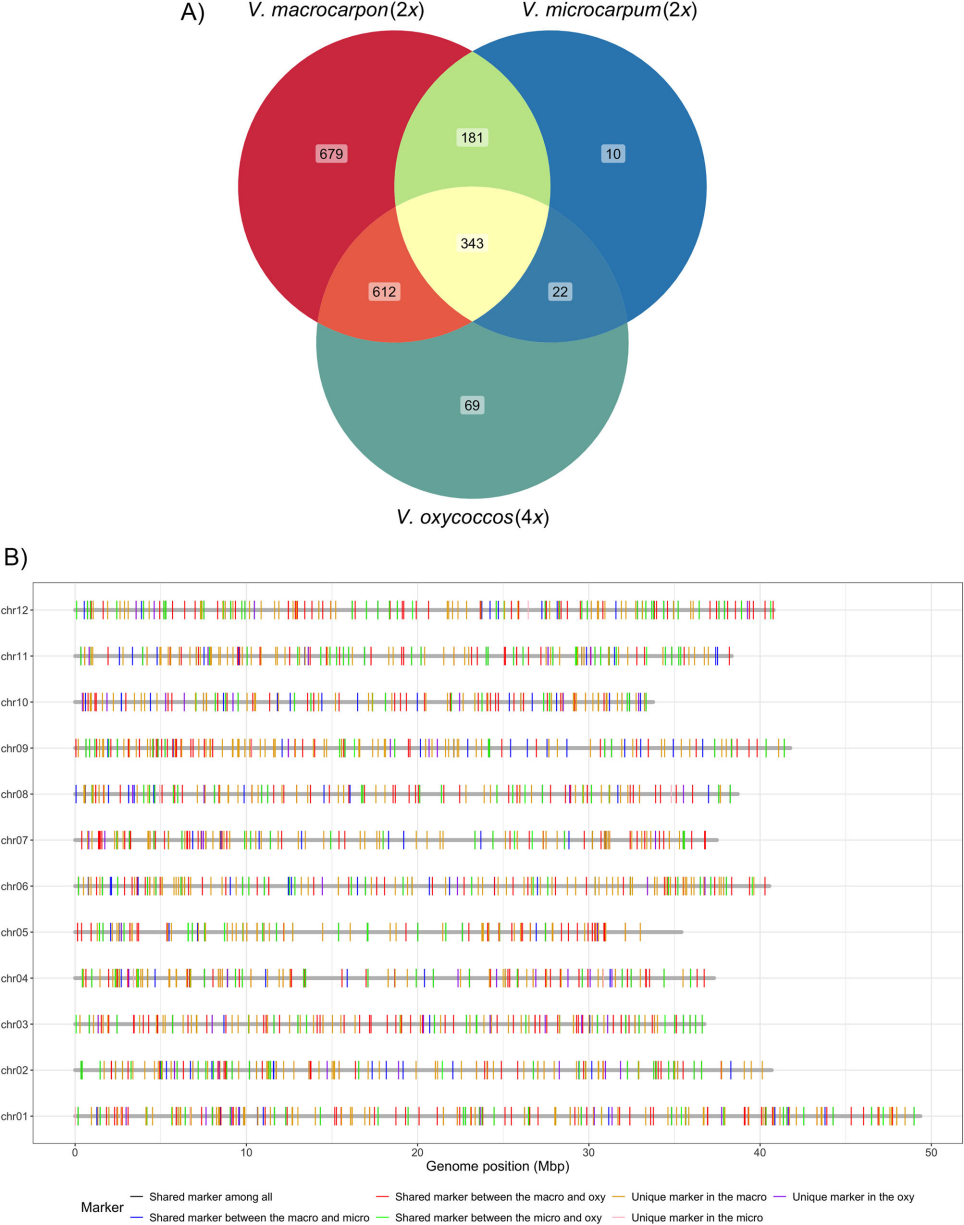
(A) Venn diagram showing marker overlap for the three species and (B) marker distribution plot of the shared and unique informative markers across the 12 cranberry chromosomes. In the legend, “macro” refers to *V. macrocarpon*, “micro” to *V. microcarpum*, and “oxy” to *V. oxycoccos*.

A total of 2540 markers were amplified in the diploid cranberry accessions (*V. macrocarpon* and *V. microcarpum*), of which 31 were not amplified in the autotetraploid cranberry accessions (*V. oxycoccos* and colchicine‐generated *V. macrocarpon*). Conversely, 2510 markers were amplified in the autotetraploid cranberry accessions, with one marker not amplified in the diploid cranberry accessions. After dosage calling and SNP filtering, 1815,556, and 1046 informative markers were retained for the *V. macrocarpon* (2*x*), *V. microcarpum* (2*x*), and *V. oxycoccos* (4*x*) accessions, respectively. A total of 343 markers were shared among all three species, along with 679 unique markers identified in the *V. macrocarpon* (2*x*), 10 in the *V. microcarpum* (2*x*), and 69 in the *V. oxycoccos* (4*x*) (Figure 3A). Markers were evenly distributed across the 12 cranberry chromosomes (Figure 3B).

**FIGURE 4:**
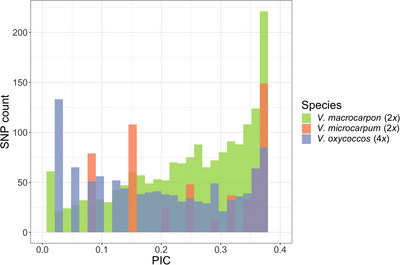
Polymorphism information content (PIC) values of 1815, 556, and 1046 informative markers in the *V. macrocarpon* (2*x*), *V. microcarpum* (2*x*), and *V. oxycoccos* (4*x*), respectively. SNP, single‐nucleotide polymorphism.

PIC values were calculated for each of the three species (Figure 4, Table S8). In the *V. macrocarpon* (2*x*), PIC values of 1815 markers spanned from 0.005 to 0.375, with an average of 0.239. In the *V. microcarpum* (2*x*), PIC values of 556 markers ranged from 0.083 to 0.375, with an average of 0.261. In the *V. oxycoccos* (4*x*), PIC values of 1046 markers spanned from 0.027 to 0.375, with an average of 0.2. PIC values of the 343 shared markers in the *V. macrocarpon* (2*x*), *V. microcarpum* (2*x*), and *V. oxycoccos* (4*x*) showed similar ranges to those of all informative markers within each corresponding species. Among the 343 shared markers, 26 showed PIC values ≥0.3 in all three species, indicating a very high level of polymorphism and potential utility in linkage map construction for inter‐specific hybrids. Similarly, the PIC values of species‐specific unique markers also fell within a similar range, and 112, 6, and 15 unique markers have PIC values ≥0.3 in the *V. macrocarpon* (2*x*), *V. microcarpum* (2*x*), and *V. oxycoccos* (4*x*), respectively.

**FIGURE 5:**
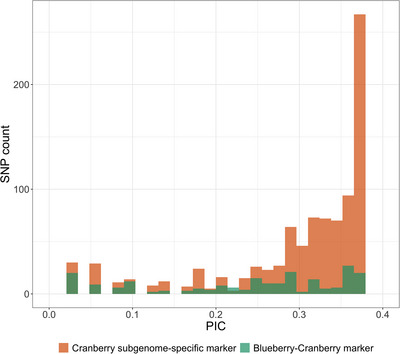
Polymorphism information content (PIC) values of 936 cranberry sub‐genome‐specific and 212 blueberry‐cranberry informative markers for the interspecific hybrids. SNP, single‐nucleotide polymorphism.

In the interspecific hybrids, a total of 1148 informative SNPs showing genome‐wide distribution (Figure S5) were retained, including 936 cranberry sub‐genome‐specific markers and 212 markers present in blueberry and cranberry genomes. The 212 blueberry‐cranberry (blue‐cran) SNPs were distributed across all 12 cranberry chromosomes, ranging from 14 to 23 SNPs on each chromosome, and could be valuable for linkage map construction in these two blue‐cran hybrid populations as the connection between the blueberry and cranberry sub‐genomes. As blueberry and cranberry are occasionally crossable due to the high proportion of unreduced gametes in *Vaccinium* (Luby et al., 1991; Lyrene et al., 2003), breeders create blueberry × cranberry or cranberry × blueberry samples as a way to move traits between the species. Future mapping populations generated from these hybrids can be used to define auto or allopolyploid segregation patterns for the blue‐cran markers. PIC values of the 936 cranberry sub‐genome‐specific markers ranged from 0.053 to 0.375, with an average of 0.257 (Figure 5, Table S9). Similarly, PIC values of the 212 blue‐cran markers ranged from 0.027 to 0.375, with an average of 0.239 (Figure 5, Table S9). These results suggested that both groups of markers had high polymorphism, indicating their potential value for interspecific hybrid population analysis.

### 3.5 Creation of linkage maps

**FIGURE 6:**
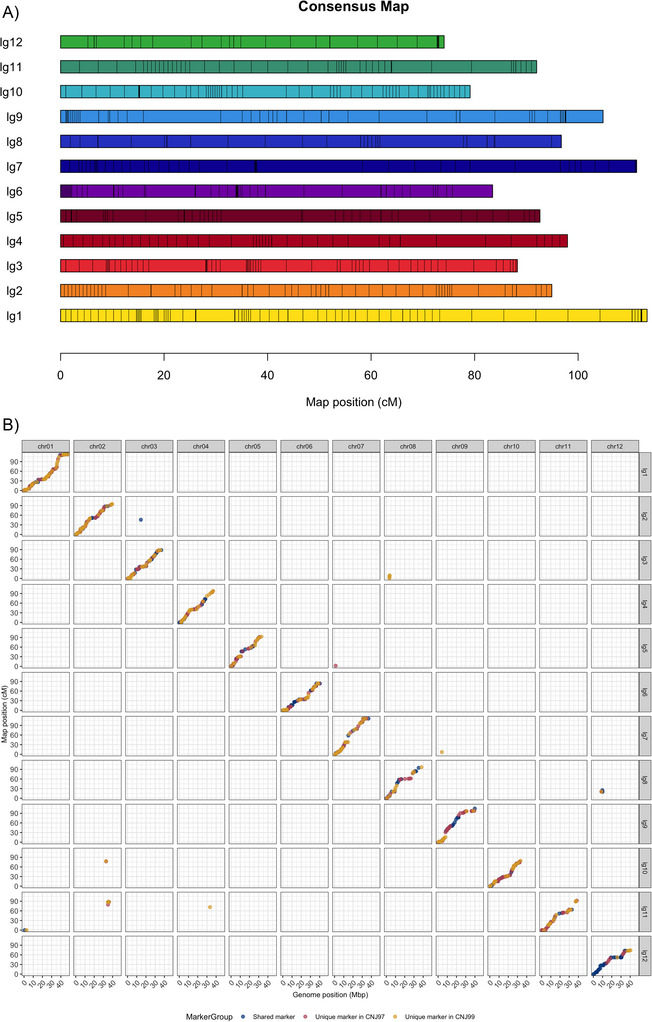
Joint genetic map of the two F_1_ populations (*n* = 124) with a shared parent (NJS98‐18). (A) Marker distribution of 764 single‐nucleotide polymorphisms (SNPs) across 12 linkage groups (lg). (B) Scatter plot showing the relationship between the genetic map position (cM) and physical genome position (Mbp) of 764 SNPs in the 12 linkage groups. Dots in the off‐diagonal squares represent SNPs in a linkage group that differ from their corresponding chromosomes in the Stevens genome. Linkage groups 1–12 correspond to the 12 chromosomes of the cranberry genome, respectively.

Individual linkage maps were constructed for two F_1_ populations, CNJ16‐45 (*n* = 64) and CNJ16‐41 (*n* = 60), using 537 and 464 markers, respectively. Of these markers, 13 and 19 were repositioned as stated in Section 2 and included into the CNJ16‐45 and CNJ16‐41 linkage maps, respectively, with seven shared repositioned markers between the two linkage maps (Table S10). Both linkage maps consisted of 12 linkage groups (LGs) with total lengths of 1128.3 cM for CNJ16‐45 and 1212.2 cM for CNJ16‐41 (Table 2; Figures S6 and S7). The linkage map for the CNJ16‐45 population exhibited higher marker density than that of the CNJ16‐41 population, with an average number of markers per centimorgan across 12 LGs of 0.48 in CNJ16‐45 compared with 0.38 in CNJ16‐41. Maximum gaps (28.7 and 26.7 cM) were observed on chromosome 8 in both populations, although they occurred between different marker intervals. A total of 237 markers were shared between these two individual linkage maps, accounting for 44.1% and 51.1% of the total markers in the linkage maps for CNJ16‐45 and CNJ16‐41, respectively. Given this high proportion of shared markers in the two linkage maps, a joint map (Figure 6A) was constructed based on shared haplotypes (Mollinari & Garcia, 2019) from the common parent, NJS98‐18, to increase marker density. The joint map included all 764 markers from the two individual maps with a similar total map length of 1128.7 cM (Table 2; Table S10). Lengths of individual LGs showed considerable variation, ranging from 74.1 to 113.3 cM, with an average of 94.1 cM. Overall, the marker density increased to 0.68 markers per centimorgan in the joint map, with the highest density of 0.87 markers per centimorgan on LG10 and the lowest of 0.47 on LG8. On average, the maximum gaps across the 12 LGs were reduced by 32.34% and 36.68% in CNJ16‐45 and CNJ16‐41, respectively (Table 3). When comparing the genetic map with the physical map, there are clear plateaus in most of the chromosomes where the centimorgan does not increase but the mega basepair does (Figure 6B). These indicated regions where recombination is repressed, most likely the centromeres and peri‐centromeric regions. The overall distribution of markers across the 12 LGs was well‐balanced, suggesting comprehensive genome coverage and providing a robust framework for future QTL mapping and MAS in cranberry breeding program.

**TABLE 3:** Summary of linkage maps for the CNJ16‐45, CNJ16‐41 population, and the joint map.

		Map length (cM)			Markers/cM			Total markers			Maximum gap (cM)		
LG	Chr	CNJ16‐45	CNJ16‐41	Joint	CNJ16‐45	CNJ16‐41	Joint	CNJ16‐45	CNJ16‐41	Joint	CNJ16‐45	CNJ16‐41	Joint
LG1	1	122.1	108.1	113.3	0.48	0.5	0.81	58	54	92	16.4	15.4	7.5
LG2	2	93.8	104.6	94.9	0.53	0.39	0.77	50	41	73	11.6	22.6	5.8
LG3	3	104.1	102.1	88.3	0.44	0.45	0.79	46	46	70	15.4	14.9	11
LG4	4	93.8	103.7	98.0	0.35	0.33	0.54	33	34	53	26.4	9.6	9.5
LG5	5	91.2	97.6	92.6	0.45	0.33	0.59	41	32	55	21.2	16.9	15.5
LG6	6	88.9	102	83.5	0.55	0.45	0.86	49	46	72	12.7	22.7	9.6
LG7	7	100.8	110.8	111.3	0.42	0.40	0.59	42	44	66	17.6	17.9	20.4
LG8	8	88.2	112.1	96.8	0.39	0.27	0.47	34	30	45	28.7	26.7	16.2
LG9	9	106.9	101.7	104.8	0.46	0.32	0.58	49	32	61	21.1	25.5	14.9
LG10	10	81.9	83.3	79.1	0.62	0.43	0.87	51	36	69	13.9	10.6	8.4
LG11	11	87.9	103.4	92.0	0.50	0.30	0.69	44	31	63	14.4	23.4	7.7
LG12	12	68.7	82.8	74.1	0.58	0.46	0.61	40	38	45	7.2	22.2	7.4
Total map		1128.3	1212.2	1128.7	0.48	0.38	0.68	537	464	764	28.7	26.7	20.4

*Note*: CNJ16‐45 population: reciprocal crosses of NJS98‐18 and CNJ97‐105‐4. CNJ16‐41 population: reciprocal crosses of NJS98‐18 and CNJ99‐9‐96.

Abbreviation: LG, linkage group.

### 3.6 Alignment of the cranberry 3K loci against the “Ben Lear” genome

Of the 3059 loci in the cranberry DArTag panel, 2485 (81%) loci were successfully aligned to the “Ben Lear” v1.0 genome. Their physical positions in the “Ben Lear” genome are provided in Table S11. Among these 2485 loci, 59 were found to be located on a different chromosome from their genomic positions within the “Stevens” genome (Table S11). These 59 loci are distributed across all 12 chromosomes, with the number of loci ranging from 1 to 19 on each chromosome. Most of 59 loci from three genomic regions (chromosome 2: 32–37 Mbp, *n* = 19; chromosome 7: 35–36.1 Mbp, *n* = 7; chromosome 12: 8–10 Mbp, *n* = 14) aligned with different chromosomes in the “Ben Lear” genome (chromosome 10 and 11, 5, and 8, respectively). All repositioned loci (*n* = 28) identified based on recombination frequencies were found among 59 loci, with 25 incorporated into the joint linkage map.

## 4 DISCUSSION

The cranberry 3K DArTag panel (CranDArTagBICUv1) is now available and open for any researcher or breeder to order through DArT (https://www.diversityarrays.com/). This platform was developed in conjunction with the resequencing and pangenome work performed by the Vaccinium Coordinated Agricultural Project (Yocca et al., 2023). The panel was designed to produce 81‐bp sequencing reads. Raw data in FASTQ format can be requested, as can the MADC file, which contains the read counts of each microhaplotype in each sample. The cranberry 3K DArTag panel demonstrated high amplification rates, with an average missing rate ranging from 12.3% to 36% at the sample level across the F_1_ populations, the diploid accessions, the autotetraploid accessions, and the *V. oxycoccos* × *V. corymbosum* interspecific hybrids. The high detection rate makes this panel suitable for genetic map construction, MAS, genome‐wide association mapping, reconstruction of recombination patterns, allele dosage estimation, and parental confirmation in North American cultivated cranberry accessions, with some limited application in other *Vaccinium* species and hybrids. The efficacy of the panel on breeding materials outside of North America has not been tested at this time.

Cranberry cultivars were also genotyped on the blueberry 3K DArTag panel (BberryDArTagBICU), where they exhibited a moderate amplification rate (average missing rate of 59.6%) but low polymorphism rate (201 out of 1588 amplified markers), suggesting the blueberry panel failed to adequately capture the genetic diversity in cranberry accessions. In contrast, the 3K cranberry DArTag panel demonstrated significantly lower average sample‐level missing rates across all cranberry accessions compared to the blueberry marker panel, reflecting its superior amplification performance for cranberry. This improved performance was expected, as the cranberry panel was specifically designed using cranberry samples. In addition, all the interspecific hybrid samples showed similar genome‐wide read count patterns with their cranberry parents (Figure 2), indicating that only the cranberry sub‐genomes in the hybrid samples were amplified well on the cranberry marker panel. However, the cranberry 3K DArTag panel showed a low amplification rate for two blueberry samples (missing rate 89.1% and 89.4%), indicating limited marker transferability to blueberry, likely due to the absence of target sequences in blueberry and/or polymorphism in primer binding sites. Collectively, despite being in the same genus and crossable, blueberry and cranberry likely contain enough sequence divergence that targeted amplicon marker panels need to be species‐specific for routine breeding work for each crop.

Compared to other cranberry species, the cranberry 3K DArTag panel showed the best average amplification rate (83.4%) in cultivated *V. macrocarpon* cranberries, followed by its hybrids that inherited partial genomic compatibility from the cranberry parent *V. macrocarpon*, reflecting the ascertainment bias we built into the cranberry DArTag panel to ensure high efficacy in *V. macrocarpon* cranberries, and some primer annealing sites might contain genetic variations in its hybrids. Although the genetic distance from *V. macrocarpon* could affect this marker panel's amplification ability in other cranberry species, their sample‐level missing rates remained below 36%, suggesting this marker panel's broader applicability across other cranberry species.

Detecting polymorphisms is crucial for selecting molecular markers in genetic studies (Serrote et al., 2020). Markers with high polymorphism rates can reduce the time and cost required for genotyping, enhancing the efficiency of genetic diversity evaluation and cultivar identification. PIC values can reflect the levels of genetic information by the allelic diversity of a locus (Botstein et al., 1980). In this study, the average PIC value exceeded 0.2 for *V. macrocarpon* (2*n* = 2*x*), *V. microcarpum* (2*n* = 2*x*), and *V. oxycoccos* (2*n* = 4*x*), indicating substantial allelic diversity within these species. The observed PIC values suggest significant genetic polymorphism across the examined *Vaccinium* taxa. There were 343 shared markers among the three species, of which 26 shared markers retained very high PIC values (≥0.3) in all the three species. These consistently polymorphic markers across different species represent a valuable resource for genomic studies and breeding applications that involve both diploid and autotetraploid cranberry accessions. Additionally, unique polymorphic markers in each species suggested the potential of markers to specific species to ensure informativeness in genomic studies.

While the “Stevens” v1.0 reference genome (Diaz‐Garcia et al., 2021) was used to develop the cranberry 3K DArTag panel, the “Ben Lear” reference genome is often used frequently in the cranberry breeding community. Therefore, an alignment for the 3K loci was performed against the “Ben Lear” v1.0 genome (Kawash et al., 2022), which revealed potential discrepancies between the two reference genome sequences. A total of 59 loci showed chromosome mismatches between the two reference genomes. Notably, all markers that were repositioned based on recombination frequencies during linkage map construction of the two F_1_ populations were found to be among these chromosome‐mismatched loci. The comparison between cranberry reference genomes provided additional support for the repositioning of markers, reinforcing the importance of recombination fraction‐based adjustments for improving marker accuracy and genomic analyses, as well as correcting errors in genome assemblies. Ultimately, 25 of the 28 repositioned markers were included in the joint map from the two individual F_1_ population maps. The incorporation of these repositioned markers in the joint map provides additional supporting evidence for the improved quality of the “Ben Lear” genome assembly relative to “Stevens.” While these findings suggested enhanced reliability, further validation may be beneficial for comprehensive downstream genomic studies.

The DArTag assay can be processed from gDNA or tissue at a cost of approximately 15 dollars per sample for the genotyping project, with returned genotyping data available in approximately 3–4 weeks. The genotyping data report comprises allele dose calls and raw data, with custom report formats available upon request. One benefit that DArTag has over fixed array platforms is the ability to update and improve the panel as required over time. The panel is a pool of 3059 oligos, one per locus, which is used to generate the sequencing libraries from the assayed material. Because the pool is created from individual oligo stocks, the removal of suboptimal loci or the addition of new loci can be easily done by creating a new pool. Independently, as new significant QTL markers and/or markers specific to other germplasm are detected, they can be included in the next iterations of the panel. DArT offers re‐pooling services once per year at low or no cost, but more frequent requests could result in labor surcharges being applied (Andrzej Kilian, personal communication, 2022). Researchers interested in initiating projects with DArT are encouraged to contact DArT directly for consultation.

We chose to create a panel of 3K loci due to cost and technical reasons; however, smaller, complementary panels can be created at lower up‐front and downstream usage costs. The addition of a complementary 3K panel would nearly double the cost of genotyping per sample but would result in more granular genotyping data.

## AUTHOR CONTRIBUTIONS

**Shufen Chen**: Formal analysis; investigation; software; validation; visualization; writing—original draft; writing—review and editing. **Meng Lin**: Data curation; formal analysis; investigation; methodology; software; supervision; validation; visualization; writing—original draft; writing—review and editing. **Cristiane H. Taniguti**: Formal analysis; software; validation; visualization; writing—review and editing. **Xuemei Tang**: Formal analysis; investigation; validation; visualization; writing—review and editing. **Fernando de la Torre**: Resources; validation; writing—review and editing. **Gina M. Sideli**: Data curation; resources; writing—review and editing. **Massimo Iorizzo**: Resources; writing—review and editing. **Patrick P. Edger**: Resources; writing—review and editing. **Jeffrey Neyhart**: Conceptualization; data curation; funding acquisition; resources; validation; writing—review and editing. **Juan Zalapa**: Conceptualization; data curation; funding acquisition; resources; validation; writing—review and editing. **Nahla Bassil**: Conceptualization; data curation; funding acquisition; resources; writing—review and editing. **Kasia Heller‐Uszynska**: Methodology; resources. **Dongyan Zhao**: Data curation; methodology; software; supervision; validation; visualization; writing—review and editing. **Craig T. Beil**: Project administration; supervision; writing—review and editing. **Moira J. Sheehan**: Conceptualization; funding acquisition; methodology; project administration; supervision; writing—review and editing.

## CONFLICT OF INTEREST STATEMENT

The authors declare no conflicts of interest.

## Supporting information




**Supplemental Figure S1**. A) Filters and criteria applied to create the cranberry 3K DArTag marker panel. M, millions; K, thousands; B) Distribution of the 3059 DArTag loci across the cranberry genome. Each red vertical line represents one of 3059 loci in physical position on the 12 chromosomes (grey bars).
**Supplemental Figure S2**. Principal component analysis (PCA) plot for the F_1_ population. CNJ16‐45: F_1_ progeny of reciprocal crosses of NJS98‐18 & CNJ97‐105‐4; CNJ16‐41: F_1_ progeny of reciprocal crosses of NJS98‐18 & CNJ99‐9‐96.
**Supplemental Figure S3**. Sample‐level missing rate of the tested materials for the 3K blueberry DArTag panel. F_1_ population includes CNJ16‐41(reciprocal crosses of NJS98‐18 & CNJ99‐9‐96), CNJ16‐45 (reciprocal crosses of NJS98‐18 & CNJ97‐105‐4), and their parents (NJS98‐18, CNJ99‐9‐96, and CNJ97‐105‐4).
**Supplemental Figure S4**. The ‘B’ allele frequency (BAF) and raw read count ratio histograms of colchicine‐created autotetraploid accessions using Qploidy. The values around 0, 0.5, and 1 are expected for a diploid sample as they present a single heterozygous class. For tetraploids, values around 0, 0.25, 0.5, 0.75 and 1 are expected since they present three possible heterozygous classes with dosages 1, 2, and 3.
**Supplemental Figure S5**. Distribution of 1148 informative markers across the 12 cranberry chromosomes in the interspecific hybrids.
**Supplemental Figure S6**. Genetic map of the CNJ16‐45 population (n = 64). A) Marker distribution of 537 single‐nucleotide polymorphisms (SNPs) across 12 linkage groups (lg). B) Scatter plot showing the relationship between the genetic map position (cM) and physical genome position (Mbp) of 537 SNPs in the 12 linkage groups. Dots in the off‐diagonal squares represent SNPs in a linkage group that is different from its corresponding chromosome in the Stevens genome. Linkage groups 1 to 12 correspond to the 12 chromosomes of the cranberry genome, respectively.
**Supplemental Figure S7**. Genetic map of the CNJ16‐41 population (n = 60). A) Marker distribution of 464 single‐nucleotide polymorphisms (SNPs) across 12 linkage groups (lg). B) Scatter plot showing the relationship between the genetic map position (cM) and physical genome position (Mbp) of 464 SNPs in the 12 linkage groups. Dots in the off‐diagonal squares represent SNPs in a linkage group that is different from its corresponding chromosome in the Stevens genome. Linkage groups 1 to 12 correspond to the 12 chromosomes of the cranberry genome, respectively.

**Supplemental Table S1**. Population composition of the testing materials for the 3K blueberry and cranberry DArTag panels.
**Supplemental Table S2**. Minor allele frequency of the Blueberry 3K DArTag panel in a cranberry population of 347 accessions estimated using read counts.
**Supplemental Table S3**. A set of 53 cranberry accessions used to create the cranberry 3K DArTag panel.
**Supplemental Table S4**. 353 marker loci removed from the raw “missing allele discovery count” (MADC) file for simplifying variant calling from partially overlapping microhaplotypes.
**Supplemental Table S5**. Sample‐based missing rate of the testing materials for the 3K blueberry and the 3K cranberry DArTag panels and sample ploidy level based on pedigree and Qploidy test.
**Supplemental Table S6**. Marker‐based missing rate of the 3K Cranberry DArTag panels across all testing samples and in each tested sample panel using genotype data points with > 10 reads for Ref + Alt haplotypes.
**Supplemental Table S7**. The first three principal components from the principal component analysis for 374 cranberry samples using read depth information.
**Supplemental Table S8**. Polymorphism information content (PIC) values for polymorphic SNPs in the *V. macrocarpon* (2x), *V. microcarpum* (2x), and *V. oxycoccos* (4x) accessions.
**Supplemental Table S9**. Polymorphism information content (PIC) values for informative SNPs in the blueberry × cranberry or cranberry × blueberry hybrids.
**Supplemental Table S10**. Marker information in the linkage map for the joint map, and each of the two individual maps.
**Supplemental Table S11**. Alignment results of the 3059 DArTag loci to ‘Stevens’ and ‘Ben Lear' cranberry reference genomes using 101 bp (± 50 bp) sequence containing target SNPs.

**Supplemental Data 1**. The “missing allele discovery count” (MADC) file for 372 cranberry samples and 2706 marker loci.

## Data Availability

The code and data are available in our GitHub repository (https://github.com/Breeding‐Insight/Cranberry_DArTag_Panel_paper/blob/main/Supplemental%20Data%201.xlsx).
