# Supplementary material for: A public mid‐density genotyping platform for cultivated cranberry (Vaccinium macrocarpon Aiton)
Source: Plant Genome. 2025 Oct 8;18(4):e70118. doi: 10.1002/tpg2.70118 (PMC12505202; doi:10.1002/tpg2.70118)
Supplement: Supplementary file 1 [file TPG2-18-e70118-s001.docx]

**Supplemental Figure S1.** A) Filters and criteria applied to create the cranberry 3K DArTag marker panel. M, millions; K, thousands; B) Distribution of the 3059 DArTag loci across the cranberry genome. Each red vertical line represents one of 3059 loci in physical position on the 12 chromosomes (grey bars).

A

B

**Supplemental Figure S2.** Principal component analysis (PCA) plot for the F_1_ population. CNJ16-45: F_1_ progeny of reciprocal crosses of NJS98-18 & CNJ97-105-4; CNJ16-41: F_1_ progeny of reciprocal crosses of NJS98-18 & CNJ99-9-96.

**Supplemental Figure S3.** Sample-level missing rate of the tested materials for the 3K blueberry DArTag panel. F_1_ population includes CNJ16-41(reciprocal crosses of NJS98-18 & CNJ99-9-96), CNJ16-45 (reciprocal crosses of NJS98-18 & CNJ97-105-4), and their parents (NJS98-18, CNJ99-9-96, and CNJ97-105-4).

**Supplemental Figure S4.** The ‘B’ allele frequency (BAF) and raw read count ratio histograms of colchicine-created autotetraploid accessions using Qploidy. The values around 0, 0.5, and 1 are expected for a diploid sample as they present a single heterozygous class. For tetraploids, values around 0, 0.25, 0.5, 0.75 and 1 are expected since they present three possible heterozygous classes with dosages 1, 2, and 3.

**Supplemental Figure S5.** Distribution of 1148 informative markers across the 12 cranberry chromosomes in the interspecific hybrids.

**Supplemental Figure S6.** Genetic map of the CNJ16-45 population (n = 64). A) Marker distribution of 537 single-nucleotide polymorphisms (SNPs) across 12 linkage groups (lg). B) Scatter plot showing the relationship between the genetic map position (cM) and physical genome position (Mbp) of 537 SNPs in the 12 linkage groups. Dots in the off-diagonal squares represent SNPs in a linkage group that is different from its corresponding chromosome in the Stevens genome. Linkage groups 1 to 12 correspond to the 12 chromosomes of the cranberry genome, respectively.

A

B

**Supplemental Figure S7.** Genetic map of the CNJ16-41 population (n = 60). A) Marker distribution of 464 single-nucleotide polymorphisms (SNPs) across 12 linkage groups (lg). B) Scatter plot showing the relationship between the genetic map position (cM) and physical genome position (Mbp) of 464 SNPs in the 12 linkage groups. Dots in the off-diagonal squares represent SNPs in a linkage group that is different from its corresponding chromosome in the Stevens genome. Linkage groups 1 to 12 correspond to the 12 chromosomes of the cranberry genome, respectively.

A

B
